# Supplemental Cardioplegia Immediately before Graft Implantation may Improve Early Post-Transplantation Outcome

**DOI:** 10.3389/fsurg.2014.00046

**Published:** 2014-11-28

**Authors:** Hendrik T. Tevaearai Stahel, Darja Unger, Juerg Schmidli, Brigitta Gahl, Lars Englberger, Alexander Kadner, Balthasar Eberle, Paul Mohacsi, Thierry P. Carrel

**Affiliations:** ^1^Clinic for Cardiovascular Surgery, Bern University Hospital (Inselspital), University of Bern, Bern, Switzerland; ^2^Department of Anaesthesiology and Pain Medicine, Bern University Hospital (Inselspital), University of Bern, Bern, Switzerland; ^3^Department of Cardiology, Bern University Hospital (Inselspital), University of Bern, Bern, Switzerland

**Keywords:** cardiac transplantation, cardioplegia, cardiac surgery outcome, organ

## Abstract

**Background:** Preservation of cardiac grafts for transplantation is not standardized and most centers use a single administration of crystalloid solution at the time of harvesting. We investigated possible benefits of an additional dose of cardioplegia dispensed immediately before implantation.

**Methods:** Consecutive adult cardiac transplantations (2005–2012) were reviewed. Hearts were harvested following a standard protocol (Celsior 2L, 4–8°C). In 2008, 100 ml crystalloid cardioplegic solution was added and administered immediately before implantation. Univariate and logistic regression analyses were used to investigate risk factors for post-operative graft failure and mid-term outcome.

**Results:** A total of 81 patients, 44 standard (“Cardio^−^”) vs. 37 with additional cardioplegia (“Cardio^+^”) were analyzed. Recipients and donors were comparable in both groups. Cardio^+^ patients demonstrated a reduced need for defibrillation (24 vs. 48%, *p* = 0.03), post-operative ratio of CK-MB/CK (10.1 ± 3.9 vs. 13.3 ± 4.2%, *p* = 0.001), intubation time (2.0 ± 1.6 vs. 7.2 ± 11.5 days, *p* = 0.05), and ICU stay (3.9 ± 2.1 vs. 8.5 ± 7.8 days, *p* = 0.001). Actuarial survival was reduced when graft ischemic time was >180 min in Cardio^−^ but not in Cardio^+^ patients (*p* = 0.033). Organ ischemic time >180 min (OR: 5.48, CI: 1.08–27.75), donor female gender (OR: 5.84, CI: 1.13–33.01), and recipient/donor age >60 (OR: 6.33, CI: 0.86–46.75), but not the additional cardioplegia or the observation period appeared independent predictors of post-operative acute graft failure.

**Conclusion:** An additional dose of cardioplegia administered immediately before implantation may be a simple way to improve early and late outcome of cardiac transplantation, especially in situations of prolonged graft ischemia. A large, ideally multicentric, randomized study is desirable to verify this preliminary observation.

## Introduction

Myocardial protection during harvesting, transport, and implantation of cardiac grafts is obviously a critical determinant of post-transplantation outcome. However, despite a significant improvement in results of cardiac transplantation over the last decades, the optimal preservation strategy has not yet been clearly defined. Several perfusates have been proposed; the currently most commonly used being the HTK (Bretschneider, Custodiol), the St-Thomas (Plegisol), and the University of Wisconsin (Belzer) solutions, all developed in the 70s, as well as the more recently developed Celsior solution (1). None of them has clearly emerged as significantly better than the other, thus leaving transplantation surgeons at liberty to select or adapt their own preservation strategy (2–4).

In a few publications, authors have described, in their methods, the adjunction of complementary doses of cardioplegic solutions administered at the time of implantation (5, 6). However, these various strategies have not been systematically evaluated (7). Therefore in the current analysis, we aimed at exploring the possible benefit of administrating an additional dose of cardioplegic solution just before starting with the implantation of the graft in terms of myocardial protection and early and mid-term post-operative outcomes.

## Materials and Methods

The study investigated a consecutive series of adult patients undergoing orthotopic cardiac transplantation at a tertiary referral cardiac surgery unit in Switzerland between January 1st 2005 and August 31st 2012. Patients were identified from a prospectively maintained institutional registry (Intellect 1.6.5, Dendrite Clinical Systems, Henley-on-Thames, UK). All patients provided informed consent for anonymized observational analyses of their data at the time of surgery and the local ethical committee approved the present investigation as part of our regular institutional quality audit.

### Study design and outcome measures

Analytic strategy was agreed before data were inspected. The main analysis focused on significant association between additional administration of a single dose of crystalloid cardioplegia immediately before graft implantation and post-reperfusion signs of myocardial damage (including the ratio of cardiac enzymes CK-MB/CK and the rate of spontaneous return to sinus rhythm), and post-operative clinically relevant in-hospital complications [including acute graft failure, the development of multiple organ failure (MOF), early mortality], and mid-term survival. We defined acute graft failure as the need to implant an IABP, ECMO, or an assist device (8). Support with any high-dose inotrope, inodilator, vasopressor, and/or pulmonary vasodilator drug was used as marker of insufficient cardiac output or tissue perfusion despite fluid resuscitation.

Secondary analyses aimed at defining independent predictors of post-operative acute graft failure.

### Surgery, myocardial protection, and anesthesia

The surgical procurement and myocardial preservation protocol were standardized, and included the infusion under low pressure of 2000 ml cold (4–8°C) Celsior solution. Celsior solution was used for cold storage and transport. Hearts implanted until August 2008 were not treated with a supplementary dose of cardioplegic solution (group “Cardio^−^”). Starting August 2008, an additional single antegrade coronary infusion of 100 ml cardioplegic solution (Cardioplexol™, Laboratorium Dr G. Bichsel AG, Unterseen, Switzerland) was administered immediately before graft implantation (group “Cardio^+^”). Cardioplexol™ is a low-volume (100 ml) crystalloid solution based on Kalium, Magnesium, Procaine, and Xylitol, and which is injected (in approximately 15 s) directly into the coronary arteries by the surgeon himself. Cardioplexol™ is typically used for standard cardiac surgical procedures. It is though to be directly injected into the aortic root by the surgeon himself, and typically induces a cardiac arrest within 5–8 s (9). Anesthetic protocol was standardized for all patients and included the intraoperative use of fentanyl or sufentanil, isoflurane, rocuronium, as well as propofol for post-operative sedation. Orthotopic transplantation was performed under moderate hypothermic cardiopulmonary bypass. Recipient cardiectomy and if present removal of a previously implanted assist device was performed timely with the availability of the donor heart. Transplantation technique was standardized with a majority of patients receiving bicaval anastomoses to prevent tricuspid valve insufficiency and sinus node dysfunction in the long term. During weaning and post-CPB, adrenaline and milrinone were used as first-line inotropic agents. Pulmonary vasodilation was routinely aimed for using inhaled nitric oxide, with inhaled iloprost as a backup. Noradrenaline was added to correct low systemic vascular resistance at high or normal cardiac output only. Azathioprine (AZA) (5 mg/kg) was given immediately before surgery and high-dose methylprednisolone (1000 mg) before reperfusion in the operating theater and a immunosuppressive regime was installed as follows: antithymocyte globulin (ATG) 4–5 mg/kg was initiated as induction therapy within 12 h post-transplant and continued for up to a maximum of five days tailored by CD3 cell count. All patients received a calcineurin inhibitor for early post-operative immunosuppression and for maintenance treatment in combination with prednisone (0.8 mg/kg/day tapered to 0.15 mg/kg/day within 5 months) and AZA or mycophenolate mofetil (MMF) (tacrolimus and MMF, replaced cyclosporine A and AZA on January 1, 2007). In case of severe early post-operative kidney dysfunction, CNI was tapered or replaced by a mammalian target of rapamycin (mTOR) inhibitor.

### Data source, management of missing values and definitions

All variables were available from our institutional registry, and were completed with specific data from our ICU (intubation time, ICU length of stay, post-operative inotropes, thoracic drainage) as well as with follow-up information provided by our institutional Heart Failure and Transplant Outpatient Unit. Prior to analysis, data were scrutinized for completeness, plausibility, and outliers. In case of missing or obviously incorrect values, alternative data sources such as hospital and ICU records were used for data replication.

Post-operative new-onset renal insufficiency was defined by a new need for dialysis or, in patients with preoperative creatinine levels below 2 mg/dl (172 μmol/l), if the post-operative creatinine level was above 2 mg/dl and at least double the preoperative value.

### Statistical analysis

Continuous variables are summarized as mean ± SD and median. To avoid undue influence of skewed data, all comparisons of continuous variables are based on non-parametric tests (Mann–Whitney). Dichotomous variables are expressed in absolute numbers and percentages, and comparisons were made using a Chi square test. Multivariable logistic regression modeling was used to assess the influence of cardioplegia, adjusting for established influence factors on acute graft failure such as ischemic time of the heart before implantation, duration of aortic cross-clamping, donor age more than 60 years, and gender of the donor. We sequentially tested different models to avoid more than three independent variables.

Kaplan–Meier curves of overall long-term survival are presented including 95% confidence intervals. Kaplan–Meier estimates of cumulative probability of survival were calculated and compared using log-rank statistics. We used Cox proportional hazard models to compare the hazard ratios of patients with vs. without additional cardioplegia, and with global cardiac ischemia longer vs. shorter than 180 min, on overall mortality, including interaction terms for cardioplegia and ischemic time.

Since the two groups were defined by two consecutive periods, we tested the possible influence of the year of transplantation surgery on the incidence of post-operative acute graft failure in a logistic regression analysis, as well as on overall mortality in a Cox regression. No association was found and this parameter was thus eliminated from further analysis.

Finally, a propensity analysis was performed using the age of the donor, the ischemic time, and the logistic EuroSCORE to construct the propensity score. The inverse probability of treatment weighting (IPTW) was included into the analyses. Tails were trimmed at both ends of the distribution in both groups in areas of suspected residual confounding.

All tests and confidence intervals are two-sided and *P* values <0.05 are deemed to indicate statistically significant differences. All statistical analysis was performed by a biostatistician (BG) using Stata 12.0 (StataCorp, College Station, TX, USA).

## Results

### Comparison of results obtained with or without additional cardioplegia

Eighty-one adult patients were transplanted during the observation period. Forty-four were operated between January 2005 and August 2008 (group “Cardio^−^”) and did not receive an additional dose of cardioplegia, as opposed to 37 patients operated between August 2008 and August 2012 (group “Cardio^+^”) who did. Nine surgeons performed between 2 and 18 transplantations (4.4 ± 2.2, median: 5). Baseline characteristics of the study groups are summarized in Table 1A. Corresponding donor characteristics and operative data are presented in Tables 1B,C, respectively. Both groups appear mostly similar except for the rate of previous cardiac surgery, which was higher in the Cardio^+^ group (86 vs. 61%, *p* = 0.011), and the rate of transplantation for which both the recipient and donor were >60 years old (16 vs. 5%, *p* = 0.079) for the Cardio^+^ and Cardio^−^ groups, respectively (Tables 1A,B).

**Table 1:** **Characteristics of 81 HTx patients distributed in two groups depending on the use of additional cardioplegia**.

	Cardio − (without additional cardioplegia) (*n* = 44)	Cardio + (with additional cardioplegia) (*n* = 37)	*p*-Value
**(A) RECIPIENTS**			
Females (%)	13 (30%)	13 (35%)	0.591
Age (years)	50.7 ± 13.5	47.8 ± 16.5	0.919
Age >60 (%)	13 (30%)	10 (27%)	0.802
Height (cm)	171.6 ± 6.8	170.7 ± 9.0	0.794
Weight (kg)	72.6 ± 14.9	71.3 ± 14.8	0.429
BSA (m^2^)	1.8 ± 0.2	1.8 ± 0.2	0.731
BMI (kg/m^2^)	24.6 ± 4.5	24.4 ± 4.3	0.888
Serum creatinine (preop, μmol/l)	124.0 ± 63.0	132.2 ± 82.6	0.919
Dialysis (preop, %)	4 (9%)	5 (14%)	0.440
Previous cardiac surgery (%)	27 (61%)	32 (86%)	0.011
Preoperative VAD/ICD (%)	32 (73%)	28 (76%)	0.763
EuroSCORE additive	12.2 ± 4.1	9.4 ± 3.2	0.068
EuroSCORE logistic	36.2 ± 24.9 (30.5)^a^	20.5 ± 15.8 (16.0)^a^	0.054
LVEF (%)	20.6 ± 11.0	25.4 ± 14.8	0.210
Urgency or emergency (%)	41 (93%)	32 (86%)	0.314
Dilatative cardiomyopathy (%)	39 (89%)	35 (95%)	0.342
Ischemic cardiomyopathy (%)	5 (11%)	2 (5%)	0.342
PHT (>150 dynes/s/cm^5^)	19 (43%)	11 (30%)	0.212
BNP	1541 ± 2950	1167 ± 2967	1.000
**(B) DONORS**			
Females (%)	15 (34%)	12 (32%)	0.875
Age (years)	45.6 ± 12.8	42.5 ± 14.6	0.216
Age >60 (%)	4 (9%)	7 (19%)	0.198
Recipient and donor age >60 (%)	2 (5%)	6 (16%)	0.079
Female donor/male recipient (%)	9 (20%)	4 (11%)	0.239
Weight (kg)	76.9 ± 10.1	76.0 ± 12.6	0.919
Donor-recipient weight discrepancy^b^	0 (0%)	0 (0%)	1.0
Inotropes (%)	26 (59%)	24 (65%)	0.861
**(C) TRANSPLANTATION**			
Graft ischemic time (min)	140 ± 44	129 ± 49	0.216
Organ ischemic time >180 min	10 (23%)	9 (24%)	0.866
Duration of surgery (min)	276 ± 72	331 ± 115	0.059
Duration of CPB (min)	139 ± 35	157 ± 46	0.150
Duration of reperfusion (min)	60 ± 25	68 ± 37	0.919
Duration of aortic cross-clamping of recipient (min)	64 ± 17	82 ± 31	0.150

*BSA, body surface area, BMI, body mass index; VAD, ventricular assist device; ICD, implantable cardioverter defibrillator; LVEF, left ventricular ejection fraction; PHT, pulmonary hypertension; BNP brain-type natriuretic peptide*.

*^a^Median*.

*^b^Donor weight is less than 30% of recipient’s weight*.

Immediate evolution after aortic declamping and myocardial reperfusion was characterized by a higher rate of spontaneous sinus rhythm in the Cardio^+^ group (48 vs. 24%, *p* = 0.030), as well as by a reduced CK-MB/CK ratio (10.1 ± 3.9 vs. 13.3 ± 4.2%, *p* = 0.001; Table 2A). Intubation time (2.0 ± 1.6 vs. 7.2 ± 11.5 days, *p* = 0.049), length of stay in ICU (3.9 ± 2.1 vs. 8.5 ± 7.8 days, *p* = 0.001) as well as the post-operative length of stay in hospital (31.3 ± 16.4 vs. 56.5 ± 59.4 days, *p* = 0.020) were shorter in the Cardio^+^ group (Table 2B).

**Table 2:** **Post-operative course and incidence of complications**.

	Cardio − (without additional cardioplegia) (*n* = 44)	Cardio + (with additional cardioplegia) (*n* = 37)	*p*-Value
**(A) IMMEDIATE POST-OP (24 h)**			
Defibrillation after aortic declamping (%)	21 (48%)	9 (24%)	0.030
CK (U/l)	617 ± 317	901 ± 470	0.003
CK-MB (U/l)	79 ± 44	86 ± 51	0.867
CK-MB/CK (%)^b^	13.3 ± 4.2 (13.0)^a^	10.1 ± 3.9 (9.5)^a^	0.001
High CK-MB/CK (>10%)	31 (70%)	16 (43%)	0.004
LDH (U/l)^b^	899 ± 283	922 ± 307	0.354
Chest tube drainage during initial six post-operative hours (ml)	704 ± 1119 (405)^a^	501 ± 538 (300)^a^	0.637
Chest tube drainage between 6 and 24 h post surgery (ml)	1485 ± 2009 (875)^a^	1028 ± 950 (709)^a^	0.637
Adrenaline during first six post-operative hours (μg/kg)	24.9 ± 25.3 (18.2)^a^	21.2 ± 19.0 (17.3)^a^	0.722
Adrenaline during first 24 h post surgery (μg/kg)	89.7 ± 110.3 (59.3)^a^	83.2 ± 71.5 (61.6)^a^	0.722
Noradrenaline during first six post-operative hours (μg/kg)	30.7 ± 135.5 (4.1)^a^	6.6 ± 10.1 (1.4)^a^	0.485
Noradrenaline during first 24 h post surgery (μg/kg)	37.2 ± 144.9 (7.7)^a^	14.8 ± 21.2 (2.9)^a^	0.903
High-dose vasopressor requirement during first 6 h^c^	11 (25%)	8 (22%)	0.554
High-dose vasopressor requirement during first 24 h^c^	7 (16%)	8 (22%)	0.569
Acute graft failure (%)^d^	4 (9%)	5 (14%)	0.528
Mortality at 24 h (%)	2 (2%)	2 (5%)	0.859
**(B) EARLY EVOLUTION (IN-HOSPITAL AND/OR 30 DAYS)**			
ICU stay (days)	8.5 ± 7.8 (4.9)^a^	3.9 ± 2.1 (3.1)^a^	0.003
ICU >5 days (%)	20 (45%)	10 (27%)	0.073
Duration of intubation (days)	7.2 ± 11.5 (2.6)^a^	2.0 ± 1.6 (1.5)^a^	0.256
Intubation >48 h	23 (52%)	13 (35%)	0.123
Cumulative chest tube drainage (ml)	4720 ± 6836 (2190)^a^	1897 ± 1551 (1290)^a^	0.041
Adrenaline (cumulative, μg/kg)	376 ± 1010 (154)^a^	177 ± 162 (120)^a^	0.724
Noradrenaline (cumulative, μg/kg)	71.1 ± 184.3 (11.8)^a^	24.5 ± 32.6 (10.3)^a^	0.818
Serum creatinine (μmol/l)	238 ± 142	240 ± 125	0.917
New-onset renal insufficiency (%)	13 (30%)	11 (30%)	0.896
New-onset dialysis (%)	8 (18%)	2 (5%)	0.083
Re-exploration (bleeding)	6 (14%)	1 (3%)	0.082
Re-exploration (infection)	6 (14%)	1 (3%)	0.077
Rejection reaction (%)	9 (20%)	4 (11%)	0.243
Multiple organ failure (%)	3 (7%)	1 (3%)	0.399
Mortality at 30 days (%)	5 (11%)	3 (8%)	0.625
for ischemic time <180 min	1	2	
for ischemic time >180 min	4	1	
Conditional survival^e^	3/42 (7%)	1/35 (2.8%)	0.398

*LDH, lactate dehydrogenase; ICU, intensive care unit*.

*^a^Median*.

*^b^Peak value during the first 24 h*.

*^c^((Adrenaline μg/kg/min × 100) + (Noradrenaline μg/kg/min × 100) + Dobutamine mg/kg/min( > 10(10)*.

*^d^Defined as the need for intra aortic balloon pump (IABP) and/or ECMO and/or VAD*.

*^e^Without first 24 h mortality*.

### Mid-term survival rate

**Figure 1:**
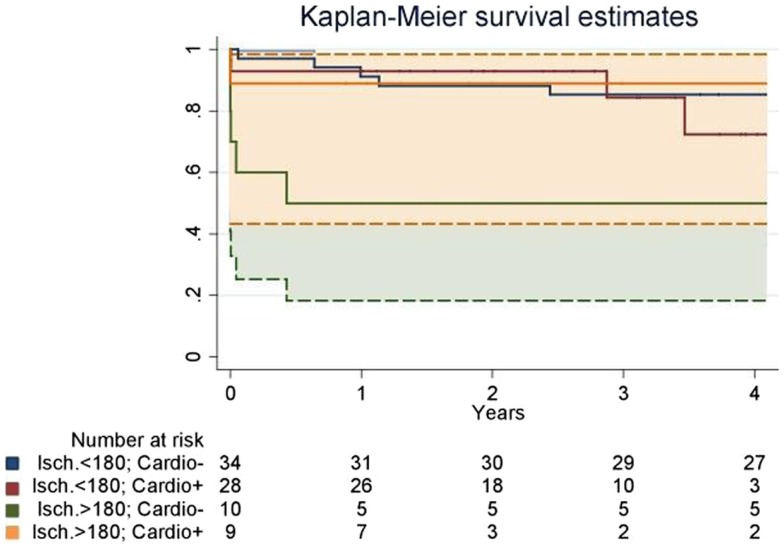
**Actuarial survival curves drawn for four subgroups defined based on an organ ischemic period superior (>180) or inferior <180) to 180 min, and the instillation of supplemental cardioplegia before graft implantation (Cardio^+^ or Cardio^−^)**. Patients who did not receive a supplemental dose of cardioplegia had a significantly decreased survival rate when ischemia was prolonged over 180 min (“Isch. >180; Cardio^−^” vs. “Isch. >180; Cardio^−^”). It was also significantly decreased as compared to patients who received a supplemental dose of cardioplegia (“Isch. >180; Cardio^−^” vs. “Isch. >180; Cardio^+^”). Dotted lines represent the confidential interval for the “Isch. >180; Cardio^−^” vs. “Isch. >180; Cardio^+^” groups.

Since both groups were consecutive, follow-up was 28.8 ± 15.0 and 60.2 ± 32.3 months in the Cardio^+^ and the Cardio^−^ groups respectively. Survival rate at 1 and 3 years were improved in the Cardio^+^ group although not statistically significant. However, analysis of subgroups based on a graft ischemic time cut-off arbitrarily set at 180 min revealed a trend for an improved survival rate in Cardio^+^ treated hearts previously exposed to graft ischemia >180 min (Table 3). Actuarial survival curves drawn for these four subgroups are displayed in Figure 1. Cardio^−^ patients with graft ischemia >180 min had a significantly decreased survival rate (Cardio^−^, *p* = 0.033). Conversely, we found no association between survival profile of Cardio^+^ patients and graft ischemic time (Figure 1).

**Table 3:** **Long-term evolution**.

	Cardio −(without additional cardioplegia)	Cardio + (with additional cardioplegia)	*p*-Value
1-year mortality (%)	8 (18%)	3 (8%)	0.203
For ischemic time <180 min	3	2	0.809
for ischemic time >180 min	5	1	0.094
3-year mortality (%)	10 (23%)	4 (11%)	0.736
For ischemic time <180 min	5	3	0.785
For ischemic time >180 min	5	1	0.393
3-year EF (%)	62 ± 5	62 ± 5	0.777
3-year LVEDD (mm)	44 ± 6	45 ± 5	0.978
3-year LVESD (mm)	28 ± 6	30 ± 4	0.181

*EF, ejection fraction; LVEDD, left ventricular end diastolic diameter; LVESD, left ventricular end systolic diameter*.

### Determination of independent predictors of post-operative acute graft failure

Immediate graft failure after reperfusion occurred in nine patients, requiring implantation of a VAD (Impella Recover, *n* = 1), veno-arterial ECMO (Medos-Deltastream, *n* = 6), and/or an IABP (*n* = 5). A univariate analysis (see Appendix) followed by a multiple logistic regression analysis identified three donor/recipient-related independent predictors (graft ischemic time; both recipient and donor age >60 years; female gender; Tables 4A and 5). Operative times were also significantly increased in this group of patients (Table 4B).

**Table 4:** **Endpoint post-operative acute graft failure: univariate analysis (selection of data)**.

	Acute graft failure		*p*-Value
	Yes	No	
	(*n* = 9)	(*n* = 72)	
**(A) DONOR**			
Organ ischemic time (min)	180 ± 38	130 ± 44	0.031
Organ Ischemic time >180 min (%)	5 (56%)	14 (19%)	0.016
Age >60 (%)	3 (33%)	8 (11%)	0.067
Recipient and Donor age >60 years (%)	3 (33%)	5 (7%)	0.012
Female (%)	6 (67%)	21 (29%)	0.024
**(B) OPERATIVE DATA**			
Cross-clamp time (min)	78 ± 24	72 ± 26	0.455
Perfusion time (min)	213 ± 48	139 ± 32	0.004
Reperfusion time (min)	117 ± 41	57 ± 22	0.004
Operation time (min)	391 ± 38	128 ± 45	0.004

*Only relevant parameters are presented. Detailed analysis is provided as Appendix*.

**Table 5:** **Endpoint post-operative assist device: logistic regression**.

	OR	95% CI	*p*
Organ ischemic time >180 min	5.48	1.08–27.75	0.040
Donor gender (female)	5.84	1.13–33.01	0.035
Recipient and Donor age >60 (%)	6.33	0.86–46.75	0.070

*Overall *R*^2^: 0.238; −2 log likelihood: −21.5; constant: 0.0037*.

*In an alternative analysis, OR (95% CI) for “organ ischemic time” entered as a continuous variable (unit = minutes) was 1.031 (1.007–1.056; *p* = 0.009)*.

### Propensity weighted analysis

Sixty-five patients remained in the IPTW analysis. The correction reduced part of the differences between the groups but was not able to fully remove all differences (data not shown). Nevertheless, with respect to the early endpoints defibrillation and CKMB/CK, the differences between the groups became even more pronounced after IPTW correction: the standardized difference increased from −0.43 to −0.6 for the defibrillation factor, and from −0.74 to −1 for CKMB/CK, indicating a large biological difference between the two groups.

## Discussion

Transplantation remains the current best option for patients suffering end-stage heart failure. However, despite large efforts to improve the rate of organ donation, the number of cardiac transplantations annually performed has not really progressed over the last two decades (11). Conversely, a continuously increasing number of patients are placed on a waiting list, a phenomenon that may be explained by the aging of the population and the higher proportion of patients surviving previous cardiac procedures, two factors which may eventually lead to a growing number of patients reaching terminal stages of cardiac insufficiency. Obviously, an increase in the number of donor organs is urgently needed, but development of strategies to further improve outcome of the recipients of those grafts is as important and necessary. Mortality after heart transplantation is still around 20, 30, and 50% after 3, 5, and 10 years, respectively (11). But in fact, the steepest drop in survival rate occurs during the first days or weeks following transplantation, and has not changed much over the last decade (11). In our current series, we also report on an early mortality rate of 11 and 8% after 30 days in the Cardio^−^ and Cardio^+^ groups, respectively (*p* = ns). As in previous reports, early post-operative mortality was mostly related to acute graft failure in our series (7/8). Interestingly, two patients, both from the Cardio^+^ group, survived the post-operative acute graft failure episode and their actuarial follow-up period is 23.3 and 15.5 months, respectively.

Survival rate over the years was demonstrated to correlate directly with the ischemic time of the graft (12–14). Especially for ischemic times of 4 h or more, mid-term and long-term success is severely compromised. This motivated the development and now testing of organ perfusion systems, such as the Transmedics’ Organ Care System (the PROCEED II trial) or the Organ Transport System’s LifeCradle, which may appear beneficial in regions where distances between the organ procurement and implantation centers are too long to allow for ischemic times <240 min. However, although promising, only little clinical experience has been gathered so that the real benefit has not been proved yet. In addition, technical requirements and costs may restrict applicability. Conversely, repeating cardioplegia administration during the ischemic period may represent a simple and attractive alternative. In Switzerland, distances between centers are short and as such, it is rare that the organ ischemic time is longer than 4 h (only one patient in our series = 241 min). For the purpose of our analysis, we therefore arbitrarily set a cut-off value to 3 h and observed that survival was significantly better among patients who received a supplementary dose of cardioplegia in situations where the organ ischemic time was prolonged over 180 min. We can however not determine how much this additional dose of cardioplegia did contribute to the improved survival since a few other factors may also have played a significant role regarding this positive evolution as for instance the change in immunosuppression in 2007.

Dispensing an additional dose of crystalloid cardioplegia at the time of implantation was not independently associated with a better outcome, in terms of post-operative acute graft failure. Nevertheless, the significant effect on several surrogates seems to indicate that this strategy may have an additional protective effect and may contribute to an overall improved post-operative evolution. We observed, for instance, that the ratio of the cardiac enzymes CK-MB/CK assessed during the first 24 h post-reperfusion was significantly reduced in situations where an additional cardioplegic dose was administered, which may indicate improved myocardial protection during the ischemic period (15–17). Also, the rate of spontaneous return to sinus rhythm was significantly increased in the Cardio^+^ group of patients, and may denote a better preserved functional integrity of the cardiac muscle and/or conduction system, as compared to the Cardio^−^ group of grafts. Finally, although the patients of both groups were in a similar preoperative condition, duration of intubation, ICU stay and post-operative duration of hospitalization were significantly reduced, which implies faster post-operative recovery.

In the current analysis, we chose to administer a new low-volume (100 ml) cardioplegic crystalloid solution, Cardioplexol™. This solution was developed in our institution, originally for procedures performed with minimized extra-corporeal circuits (MECC). Our experience with this type of cardioplegia on more than 8000 patients, which has been only partially reported to date (9), demonstrated several advantages over other more traditional approaches, including an almost immediate cardiac arrest and a prolongation of the cardioprotective effect over 45 min or more, without the need to administer a second dose, and also minimal hemodilution due to the reduced volume injected. The solution is currently undergoing evaluation in a clinical phase 3 registration study in Europe (EudraCT Number 2011-004198-10). In the current study, we chose this solution over others because of the simplicity of its administration (two 50 ml syringes of the solution are slowly injected into both coronary ostia by the surgeon). It remains to be proven whether similar results could have been reproduced with a different solution administered at the time of implantation.

Obviously, our current study has limitations, and the results must thus be interpreted with caution. The study is monocentric and retrospective and the patients belonged to two consecutive groups implying that other less measurable aspects, such as the experience of the entire team, including anesthesiologists and intensivists, may have played a role in improving our results during the second period when additional cardioplegia was used. Also, the relatively small number of patients is a limitation. Although a series of risk factors may influence the occurrence of acute graft rejection (such as the change of the post-operative maintenance immunosuppression), the statistical analysis we performed was restricted to a few parameters. In addition, the propensity score could only partially compensate the difference between the groups. Finally, and as already mentioned, the results obtained with Cardioplexol™ may not be generalizable to other cardioplegic solutions. Nevertheless, we believe the findings presented here are hypothesis-generating, and will encourage further development of protocols to improve cardioplegia in transplanted hearts. In that sense, prospective randomized, ideally multicentric, studies should be designed, especially since it might be that this simple modification of a cardioplegia protocol represents an attractive and cost-effective alternative to the use of *ex vivo* organ perfusion systems.

## Conflict of Interest Statement

Hendrik T. Tevaearai Stahel and Thierry P. Carrel are inventors of the Cardioplexol™ solution. The other co-authors declare that the research was conducted in the absence of any commercial or financial relationships that could be construed as a potential conflict of interest.

## Appendix

**Table A1:** **Characteristics of 81 HTx patients distributed in 2 groups depending on the occurrence of post-operative acute graft failure**.

	Acute graft failure		*p*-Value
	Yes	No	
	(*n* = 9)	(*n* = 72)	
**(A) RECIPIENTS**			
Females (%)	4 (44%)	22 (31%)	0.400
Age (years)	49.5 ± 16.2	49.3 ± 14.9	0.969
Age >60 (%)	3 (33%)	20 (28%)	0.727
Size (cm)	168.2 ± 5.0	171.5 ± 8.1	0.119
Weight (kg)	71.4 ± 9.9	72.1 ± 15.3	0.969
BSA (m^2^)	1.8 ± 0.1	1.8 ± 0.2	0.969
BMI (kg/m^2^)	25.2 ± 3.3	24.4 ± 4.6	0.906
Serum creatinine (μmol/l)	153.3 ± 43.8	124.5 ± 74.7	0.146
Dialysis (%)	0 (0%)	9 (13%)	0.059
Previous cardiac surgery (%)	8 (89%)	51 (71%)	0.251
Preoperative VAD/ICD (%)	5 (56%)	55 (76%)	0.179
EuroSCORE additive	10.1 ± 3.6	11.0 ± 4.0	0.789
EuroSCORE logistic	24.4 ± 20.1	29.6 ± 22.9	0.968
LVEF (%)	21.1 ± 7.0	23.0 ± 13.6	0.644
Urgency or emergency (%)	7 (78%)	66 (92%)	0.363
Dilatative cardiomyopathy (%)	8 (89%)	66 (92%)	0.780
Ischemic cardiomyopathy (%)	1 (11%)	6 (8%)	0.780
PHT (>150 dynes/s/cm^5^)	33 (33%)	27 (38%)	0.807
BNP	635 ± 365	1465 ± 2995	1.000
**(B) DONORS**			
Females (%)	6 (67%)	21 (29%)	0.024
Age (years)	46.6 ± 17.5	43.9 ± 13.2	0.969
Age >60 (%)	3 (33%)	8 (11%)	0.067
Weight (kg)	72.6 ± 9.5	77.0 ± 11.4	0.504
Recipient and donor age >60 (%)	3 (33%)	5 (7%)	0.012
Female donor/male recipient (%)	3 (33%)	10 (14%)	0.134
Weight <30% recipient (%)	0 (0%)	0 (0%)	
Inotropes (%)	6 (67%)	44 (61%)	0.634
**(C) TRANSPLANTATION**			
Organ ischemic time (min)	180 ± 38	130 ± 45	0.031
Organ ischemic time >180 min	5 (56%)	14 (19%)	0.016
Operation duration (min)	391 ± 68	290 ± 270	0.004
Perfusion time (min)	213 ± 48	139 ± 32	0.004
Reperfusion time (min)	117 ± 41	57 ± 22	0.004
Cross-clamp time (min)	78 ± 24	72 ± 26	0.455

*BSA, body surface area, BMI, body mass index; VAD, left ventricular assist device; ICD, implantable cardioverter defibrillator; LVEF, left ventricular ejection fraction; PHT, pulmonary hypertension; BNP, brain-type natriuretic peptide*.
